# Relationship between Chinese middle-aged and old couples' Confucian coping thinking and marital quality

**DOI:** 10.3389/fpubh.2022.956214

**Published:** 2022-09-23

**Authors:** Zhiguang Fan, Hanwei Wu, Min Tao, Lei Chen

**Affiliations:** ^1^Department of Education, Jilin International Studies University, Changchun, China; ^2^Department of English, Jilin International Studies University, Changchun, China; ^3^Department of Marxism, Changchun University of Chinese Medicine, Changchun, China

**Keywords:** middle-aged and old couples, responsibility thinking, pro-setback thinking, fate thinking, marital quality, the actor-partner interdependence model

## Abstract

**Objective:**

Drawing on the actor-partner interdependence model (APIM), the present study investigated the relationship between Chinese middle-aged and old couples' Confucian coping thinking and their marital quality in the hope to provide a theoretical basis for ameliorating marital quality.

**Methods:**

With 744 middle-aged and old couples as participants, the Confucian Coping Questionnaire (CCQ) and the Quality of Marriage Index (QMI) were employed to probe the relationship between responsibility thinking (RT), pro-setback thinking (PT), fate thinking (FT), and marital quality.

**Results:**

Husbands' and wives' scores in responsibility thinking and pro-setback thinking had significantly positive correlations with their own and their spouses' scores in marital quality, respectively, and husbands' and wives' scores in fate thinking had significantly negative correlations with their own and their spouses' marital quality, respectively. Husbands' responsibility thinking, pro-setback thinking, and fate thinking had a significant actor effect. Husbands' responsibility thinking and fate thinking had a significant partner effect. Wives' responsibility thinking, pro-setback thinking, and fate thinking had a significant actor effect. Wives' responsibility thinking and pro-setback thinking had a significant partner effect.

**Conclusion:**

From the perspective of dyadic relationships, the present study found that responsibility thinking and pro-setback thinking could positively predict marital quality, while pro-setback thinking could negatively predict marital quality.

## Introduction

For most people, establishing and maintaining a meaningful and positive marital relationship is an indispensable experience in their lives. Marital quality is regarded as the main indicator of marital harmony and marital stability (1). How to improve marital quality is always one of the hotspots in the marriage and family field. Marital quality is couples' subjective evaluation of their marital satisfaction and marital harmony (2). Inferior marital quality not only can negatively predict individuals' psychological health (3) but also can impair physical health (4). On the contrary, superior marital quality can be beneficial to improving marital stability (5), reducing psychological distress (6), mitigating loneliness, (7), and buffering negative emotions' adverse effects (8). Besides, related to individuals' physical activity and health behaviors, marital quality can also predict one's physical health (9). In general, individuals with higher marital quality have higher cognitive health (10), better sleep quality (11), less pain (12), and fewer disease risks (13). Moreover, low parental marital quality can also lower adolescent psychological wellbeing (14).

Coping approaches are a key predictor of marital quality (15). There might be stress, divergence, arguments, and even fights in any marriage, although a great marital relationship is expected by couples (16). The vulnerability-stress-adaptation (VSA) model assumes that the coping approaches adopted by couples in the face of daily life events and marital stress are directly associated with their subjective perception and evaluation of marriage (17). Positive emotional expressions and search for solutions can positively predict marital quality, while negative emotional expressions and avoidance of problems can negatively predict marital quality (18). On balance, coping approaches in marriage can be divided into positive and negative ones. Positive coping approaches are conducive to mitigating emotional distress between couples and enhancing the level of perceived social support for couples (19) and ameliorating marital quality (20). By contrast, negative coping approaches can be destructive to marital quality (21), and even negatively impact individuals' marital quality in a long run (22).

Coping approaches clearly show cultural characteristics, since individuals in different cultural contexts have different perceptions, evaluations, and selection of coping objectives and approaches (23). Chinese traditional culture is a composite of multiple cultures represented by Confucianism, Taoism, and Buddhism. As the mainstream culture, Confucianism has greatly contributed to Chinese people's ideology, behavioral patterns, and psychological health (24). Confucianism has a rich discussion of how individuals deal with stress and setbacks. Different from the western culture, which assumes that stress derives from external or specific life events, Confucianism regards stress as the corollary of insufficient self-cultivation and emphasizes that the ideal coping approaches should be “unity of knowledge and action,” namely, individuals constantly improve their ability to cope with stress through moral cultivation, meanwhile cultivating their morality through specific practice (25). Furthermore, cultural differences may also be shown in the outcome of coping in the Chinese and Western contexts (26).

Confucian coping thinking refers to Chinese people's mindset in coping with difficulties and setbacks under the influence of Confucian culture, which mainly comprises responsibility thinking, pro-setback thinking, and fate thinking (27). As an important component of Confucianism, responsibility thinking assumes that individuals have responsibility for themselves, others, society, and all things in the world (28). “Cultivating the self, regulating the family, governing the country, and pacifying the world” are the crucial political ideals of Confucianism. Accordingly, a person's responsibility extends from himself to the family, the country, and even the world. Family is not only the basic unit of social organization but also the hub connecting individuals and countries. Responsibility for the family is often regarded as one's most important responsibility (29). Individuals with high responsibility thinking, whether in good or bad times, tend to voluntarily take responsibility and actively handle and solve a variety of negative events. Although Confucianism agrees that setbacks can bring pain and tension, it also believes that setbacks can effectively hone oneself and benefits one's growth (30). Individuals with high pro-setback thinking pay more attention to the positive aspects of hardship and believe that the key to success is to accept, face and overcome setbacks through their own efforts (31). Moreover, fate thinking plays a vital role in Confucian culture and greatly contributes to the coping approaches of Chinese people. Individuals with high fate thinking think that their fate is predetermined so that when encountering difficulties, they have a stronger sense of powerlessness and are less likely to seek solutions to problems (27).

Although the relationship between coping approaches and marital quality has been widely demonstrated, few studies have investigated the effect of coping approaches on marital quality from the cultural perspective (32). Since individuals' coping approaches are closely associated with their cultural contexts (33), Chinese couples would inevitably show certain coping characteristics in conformity with Chinese culture. In most cases, responsibility thinking and pro-setback thinking are connected to positive psychological and behavioral outcomes, while fate thinking is connected to negative outcomes (31). According to previous studies, individuals' responsibility thinking and pro-setback thinking are positively correlated with psychological resilience and negatively correlated with anxiety and depression (26, 27). Besides, psychological resilience is considered to increase marital quality (34), whereas anxiety and depression are regarded to decrease marital quality (35). In addition, responsibility thinking and pro-setback thinking are the major constituents of positive Confucian ideology. Confucianism believes that the happiness brought by fulfilled personal needs is based on voluntarily taking responsibility and strenuously surmounting difficulties (30). Hence, individuals with high responsibility thinking and pro-setback thinking can face frustrations with more equanimity and handle the problems in marriage with more optimism, which can improve marital quality. Therefore, it seems reasonable to postulate that responsibility thinking and pro-setback thinking can positively predict marital quality, whereas fate thinking could negatively predict marital quality.

Dyadic relationships are the basic unit of interpersonal interaction (36). Since marital relationships are the closest interpersonal relationship, one party's notions and behaviors indispensably have effects on another party, which is particularly remarkable in China where interpersonal connections are highly emphasized (37). Self includes independent self and interdependent self, and Chinese people prioritize society-oriented interdependent self (38). Individuals with high interdependent self tend to regard themselves as a part of relationships, expect more to gain recognition from others, and consider the needs of others (39). Family systems theory assumes that the family can be divided into three subsystems of marital, parent–child, and sibling relationships. The same and different subsystems are interrelated and interacted (40). Since marital relationships are the most crucial subsystem, couples have strong interdependence in cognition, emotion, and behavior (41). Hence, it seems advisable to presume that husbands' or wives' Confucian coping thinking not only can affect their own marital quality but also likely affect their spouses' marital quality.

The APIM is widely employed in the analysis of dyadic data. The actor effect refers to the effect of individuals' predictor variables on their own outcome variables, while the partner effect comes to the effect of individuals' predictor variables on their partner's outcome variables (42). Unfortunately, although a multitude of studies have indicated that couples' coping approaches can predict both their own and their spouses' marital quality (32, 43), so far, no study has probed the relationship between Confucian coping thinking and marital quality using the APIM. It's noteworthy that several studies found that gender differences may exist in the partner effect of coping approaches on marital quality (44). Brandão et al. found that both husbands' and wives' dyadic coping had an actor effect on marital quality, but only husbands had a partner effect on marital quality (45). The longitudinal research also demonstrated that couples' coping approaches have different effects on their spouses' marital quality (46). Hence, it can be extrapolated that Confucian coping thinking is probably related to both one's own (the actor effect) and their spouses' marital quality (the partner effect).

According to the above analysis, several gaps in the extant literature can be crystallized. First, a paucity of studies has explored the relationship between Confucian coping approaches and marital quality, notwithstanding its cultural significance. Second, few studies have applied the APIM to probe this relationship, although it offers an effective framework. Given the existing gaps in research, we aimed to culturally investigate this relationship using the APIM. More specifically, we hypothesized that individuals' responsibility thinking and pro-setback thinking were positively correlated with their own and their spouses' marital quality, and fate thinking was negatively correlated with their own and their spouses' marital quality.

## Materials and methods

### Study design and participants

The inclusion criteria for participants are: (1) Marital length≥15 years, (2) Age≥40 (both husbands and wives) (47), and (3) Couples who volunteered for the survey and signed the informed consent. The present study was approved by the Ethics Committee of Jilin International Studies University.

A household survey was conducted by systematically trained college students among middle-aged and old couples in their hometowns. Participants were from eight provinces of China, namely, Jilin, Heilongjiang, Hebei, Henan, Shandong, Inner Mongolia, Sichuan, and Gansu. Data collection was based on convenient sampling and snowball sampling. More specifically, researchers first had a survey on their acquaintances like relatives, friends, and neighbors. Afterward, researchers requested these acquaintances to recommend new participants meeting the inclusion criteria. By the same token, these new participants were requested to provide other new participants. In this way, sample sizes were continuously expanded. It should be noted that the survey was conducted face to face.

Before the survey, participants were informed of the purpose, the way to complete the questionnaire, confidentiality, and anonymity. Besides, participants' questions about the survey were answered, and their permission was obtained. After the paper informed consent was signed, each couple got a code for matching the data. After that, participants were sent a link for the survey from researchers to complete the online questionnaire. The questionnaire was administered separately to husbands and wives in case they influence each other. If participants had difficulties in reading the questionnaire due to their low educational level or poor eyesight, researchers would read and fill out the questionnaire for them. After the survey, researchers checked the data and removed invalid questionnaires. The criteria for removal are as follows: (1) The response time of husbands/wives was too short (<120 s). (2) Both positive and negative items were responded to the same. (3) The data could not be matched.

A total of 813 husband-reported questionnaires were collected, of which, 13 questionnaires were dropped for both positive and negative items being responded to the same, 28 questionnaires were removed for response time being too short (<120 s), and then 769 questionnaires were retained. A total of 821 mother-reported questionnaires were collected, of which, 17 questionnaires were dropped for both positive and negative items being responded to the same, 37 questionnaires were removed for response time being too short (<120 s), and then 783 questionnaires were retained. Finally, 744 sets of valid data were collected. Regarding residence, 414 (55.65%) couples lived in the city, and 330 (44.35%) couples lived in the countryside. Concerning marital length, it ranged from 15 to 59 years (Mean ± SD = 29.77 ± 12.99). Regarding annual family income, 74 couples earned <10,000 yuan (9.95%), 159 couples earned between 10,000 and 30,000 yuan (21.37%), 158 earned between 30,000 and 50,000 yuan (21.24%), 181 couples earned between 50,000 and 100,000 yuan (24.33%), 90 couples earned between 100,000 and 150,000 yuan (12.10%), 55 couples earned between 160,000 and 250,000 yuan (7.39%), 22 couples earned between 250,000 and 500,000 yuan (2.95%), and 5 couples earned more than 500,000 yuan (0.67%). Regarding age, husbands aged from 40 to 79 (Mean ± SD = 55.14 ± 11.67) and wives aged from 40 to 77 (Mean ± SD = 53.65 ± 12.12).

### Measures

#### Confucian coping questionnaire

The Confucian Coping Questionnaire (CCQ) was first developed by Jing Huaibin and then revised by Yang Muzi (27). The scale consists of 11 items divided into three dimensions of responsibility thinking, pro-setback thinking, and fate thinking, scored on a Likert scale from 1 (strongly disagree) to 5 (strongly agree). Among them, items 3, 4, 7, 8, and 9 come to responsibility thinking, items 1, 5, and 11 belong to pro-setback thinking, and items 2, 6, and 10 refer to fate thinking. The sum of all item scores is the total scores. The higher scores in responsibility thinking, the more recognition of taking responsibility. The higher scores in pro-setback thinking, the more positive attitude toward setbacks, and the more identification with setbacks' benefits on one's growth. The higher scores in fate thinking, the more approval for fate thinking. Item examples in each dimension: “People should naturally take social responsibility,” “Only those experiencing many setbacks can be successful,” and “A good or bad life is determined by external and mysterious fate.” In the present study, Cronbach's alpha coefficients of responsibility thinking, pro-setback thinking, and fate thinking were 0.795, 0.718, and 0.704 for husbands, respectively, and 0.789, 0.707, and 0.705 for wives, respectively.

#### Quality of marriage index

Developed by Norton in 1983, the Chinese version of the Quality of Marriage Index (QMI) was employed (48, 49). The scale with one dimension is composed of six items. Participants answer the first five items on a 7-point scale ranging from 1 (strongly disagree) to 7 (strongly agree). The sixth item is answered on a 10-point scale ranging from 1 (extremely low) to 10 (extremely high). The sum of all item scores is the total scores, with higher scores indicating higher marital quality. Item examples: “My relationship with my partner is very stable” and “My relationship with my partner makes me happy.” In the present study, Cronbach's alpha coefficients were 0.929 for husbands and 0.927 for wives.

### Statistical analysis

Descriptive statistics and item analysis were performed using SPSS 26.0. The correlation between the variables between husbands and wives was tested using Pearson's correlation coefficient. The APIM was tested using an online free web application called APIM_SEM (50). With maximum likelihood estimation (MLE), the analyses used structural equation modeling (SEM) using the program R package lavaan (51). Three APIMs were constructed to test the effect of couples' responsibility thinking, pro-setback thinking, and fate thinking on their own and their spouses' marital quality, respectively. When examining the APIM, we set marital length, annual family income, and residence as the control variables. The actor effect refers to the effect of individuals' predictor variables on their own outcome variables, while the partner effect comes to the effect of individuals' predictor variables on their partner's outcome variables. For instance, in the present study, the husbands' actor effect refers to the predictive effect of husbands' Confucian coping thinking on their own perceived marital quality, whereas the husbands' partner effect comes to the predictive effect of wives' Confucian coping thinking on husbands' marital quality. The standard model of the APIM was saturated and just-identified. Four general dyadic patterns include the actor-only, the couple, the contrast, and the mixed patterns. In the analysis of dyadic patterns, *k-*values, used to measure dyadic patterns, are the ratio of the partner effect to the actor effect. Only when the standardized absolute values of the actor effect are higher than 0.10 and statistically significant, *k*-values can be computed. With 5,000 bootstrap iterations, the confidence interval (CI) for *k*-values was computed, and if 1, 0, or −1 was in the CI was evaluated. If 0 is in the CI, the model is the actor-only pattern; if 1 is in the CI, the model is the couple pattern; if −1 is in the CI, the model is the contrast pattern (52); if the CI is between 0 and 1, it suggests that the APIM is between the couple pattern and the actor-only pattern, called the mixed pattern (53). Although dyadic relationships are distinguished by the role (husband vs. wife), their actor and partner effects probably cannot be distinguished (54). Two steps are needed to examine whether dyad members are distinguishable or indistinguishable. First, test if the actor and partner effects can be set equal. In this step, the actor and partner effects of the dyadic data are set equal to test whether the chi-square value significantly changes. The insignificant change in the chi-square value (*p* > 0.05) indicates that the actor and partner effects as the dyad numbers are probably indistinguishable (52). Second test indistinguishable dyad members. In this step, a model with complete indistinguishability is constructed by setting equal means and variances of the causal variables, intercepts of the outcome variables, error variances, actor effects, and partner effects (55). To test if gender makes a statistically significant difference, model comparison is performed by a chi-square test between a model with distinguishable members and a model with indistinguishable members. If *p* < 0.05, it suggests that members can be statistically distinguished by gender.

## Results

### Preliminary analyses

Table 1 lists the mean and standard deviation of the scores of husbands and wives in the CCQ and the QMI. As illustrated in Table 2, the scores of husbands and wives in responsibility thinking and pro-setback thinking were significantly positively correlated with their own and their spouses' marital quality, respectively. The scores of husbands and wives in fate thinking were significantly negatively correlated with their own and their spouses' marital quality, respectively.

**Table 1:** Descriptive characteristics of the CCQ and the QMI.

**Items**	**Husband**		**Wife**	
	**M**	**SD**	**M**	**SD**
Without suffering, there will be no sheer tenacity.	3.92	1.01	3.79	1.06
Fate is a random result of various external factors.	2.78	1.31	2.74	1.19
I still feel hopeful for future even in confronting my biggest failure.	3.98	1.06	3.94	1.06
I can control how the thing is going on.	3.46	0.96	3.37	0.94
People with a smooth life won't have great success.	2.94	1.01	2.80	1.02
A good or bad life is determined by external and mysterious fate.	2.21	1.27	2.25	1.23
I always try to learn something from setbacks.	3.96	1.05	3.94	1.03
I still try to improve myself to prepare for future in the time of bad luck.	4.15	1.01	4.10	1.03
People should naturally take social responsibility.	3.99	1.08	3.93	1.09
Fate is mysterious and predetermined.	2.32	1.27	2.35	1.21
Only those experiencing many setbacks can be successful.	3.37	1.02	3.25	1.03
RT	19.53	3.83	34.92	7.44
PT	10.26	2.40	9.85	2.48
FT	7.34	2.88	7.30	3.05
We have a good marriage.	5.36	1.41	5.23	1.42
My relationship with my partner is very stable.	5.37	1.40	5.29	1.36
Our marriage is strong.	5.42	1.40	5.32	1.39
My relationship with my partner makes me happy.	5.43	1.40	5.30	1.40
I really feel like part of a team with my partner.	5.40	1.43	5.32	1.35
The degree of happiness, everything considered, in your marriage?	8.74	1.54	8.46	1.74
QMI	35.71	7.38	34.92	7.44

M, mean; SD, standard deviation; RT, responsibility thinking; PT, pro-setback thinking; FT, fate thinking; QMI, quality of marriage index.

**Table 2:** Descriptive statistics and correlations of all variables.

**Variables**	**RT**	**PT**	**FT**	**QMI**	**H-M**	**H-SD**
RT	0.30^***^	0.41^***^	−0.09^*^	0.32^***^	19.53	3.83
PT	0.43^***^	0.24^***^	0.29^***^	0.17^***^	10.26	2.40
FT	−0.12^**^	0.20^***^	0.37^***^	−0.21^***^	7.30	3.05
QMI	0.40^***^	0.18^***^	−0.20^***^	0.46^***^	35.71	7.38
W-M	19.29	9.85	7.34	34.92		
W-SD	3.80	2.48	2.88	7.44		

H, husband; W, wife; M, mean; SD, standard deviation; RT, responsibility thinking; PT, pro-setback thinking; FT, fate thinking; QMI, quality of marriage index; ^*^p < 0.05, ^**^p < 0.01, ^***^p < 0.001. Below the diagonal are the ones for husbands, above the diagonal for wives, and the correlations between husbands and wives are on the diagonal.

### Testing for APIM

**Figure 1:**
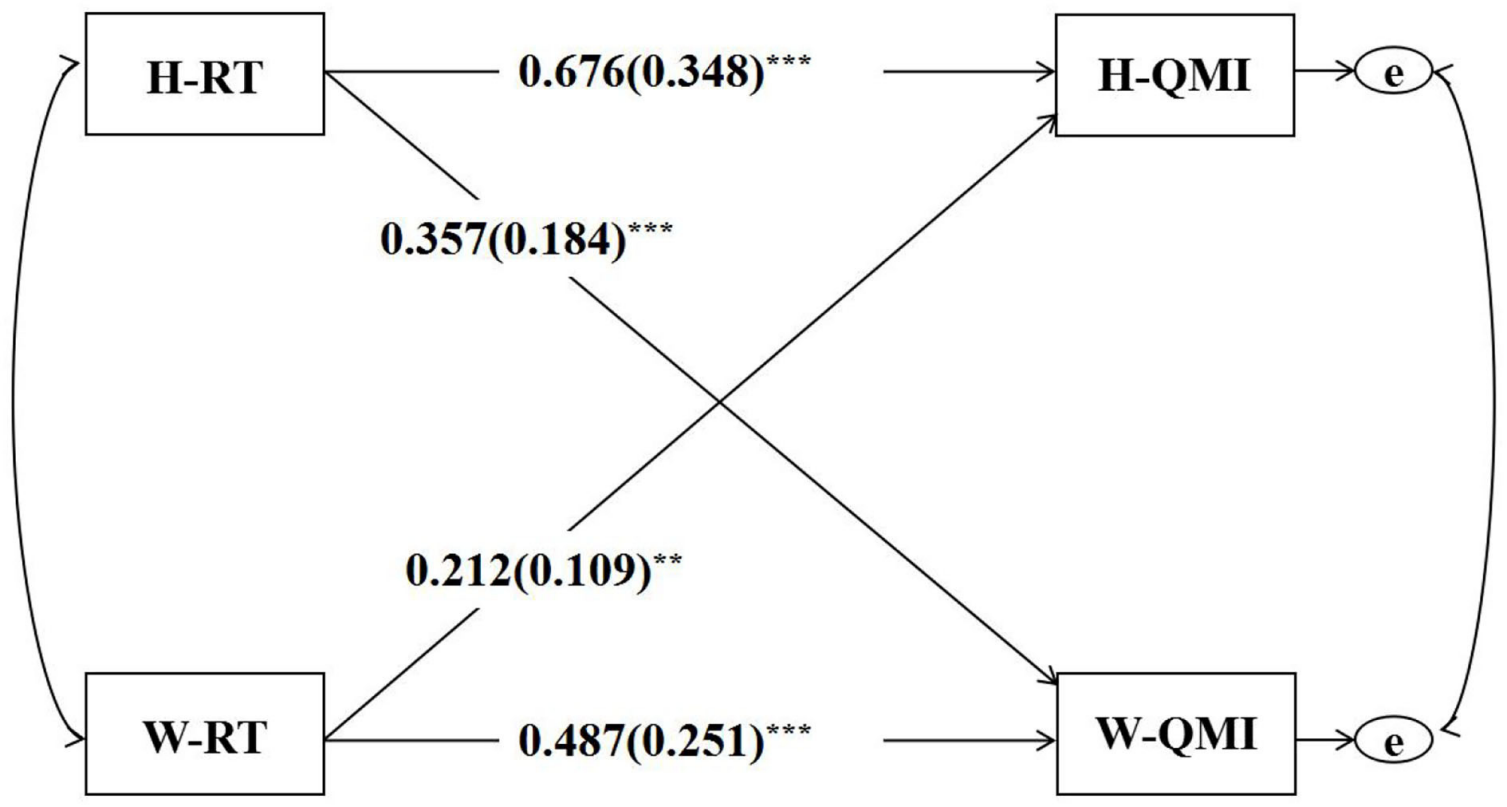
The APIM for RT's predictive effect on QMI. RT, responsibility thinking; QMI, quality of marriage index; H, husband; W, wife; **p < 0.01, ***p < 0.001.

Three APIMs for the predictive effect of responsibility thinking, pro-setback thinking, and fate thinking on marital quality were constructed, respectively (see Table 3). Specifically, the APIM for the predictive effect of couples' responsibility thinking on marital quality is illustrated in Figure 1, and the actor and partner effects for husbands and wives are presented in Table 2. When tested if the two actor effects were equal, the difference was found not to be statistically significant [*p* = 0.070, 95%CI (−0.02, 0.39)]. Additionally, when tested if the two partner effects were equal, the difference was found not to be statistically significant [*p* = 0.152, 95% CI (−0.34, 0.05)]. Husbands' *k*-value was 0.313 with a 95% CI between 0.110 and 0.571 (the CI was between 0 and 1), suggesting that the pattern was a mixed pattern. The wives' *k*-value was 0.734 with a 95% CI between 0.401 and 1.263 (1 was in the CI), suggesting the couple pattern. Besides, the two *k*-values showed no significant difference [*p* = 0.132, 95% CI (−1.04, 0.07)]. Whether the pattern was with distinguishable dyad members was further tested. According to the results, χ^2^(15) was equal to 192.21 and *p* was lower than 0.001, suggesting that the predictive effect of husbands' and wives' responsibility thinking on marital quality was statistically distinguishable dyad members by gender.

**Table 3:** Parameter estimates for paths of the APIM.

	**Effect**	**Estimate**	**Standardized effect**	**95% CI**	***p***
RT	H-Actor	0.676	0.348	[0.533, 0.824]	<0.001
	H-Partner	0.212	0.109	[0.079, 0.343]	0.002
	H-*k*	0.313		[0.110, 0.571]	
	W-Actor	0.487	0.251	[0.352, 0.626]	<0.001
	W-Partner	0.357	0.184	[0.220, 0.500]	<0.001
	W-*k*	0.734		[0.401, 1.263]	
PT	H-Actor	0.476	0.157	[0.256, 0.699]	<0.001
	H-Partner	0.108	0.036	[−0.089, 0.306]	0.285
	H-*k*	0.227		[−0.181, 0.853]	
	W-Actor	0.441	0.145	[0.236, 0.644]	<0.001
	W-Partner	0.303	0.100	[0.102, 0.511]	0.004
	W-*k*	0.686		[0.191, 1.778]	
FT	H-Actor	−0.362	−0.145	[−0.560, −0.165]	<0.001
	H-Partner	−0.225	−0.090	[−0.414, −0.041]	0.018
	H-*k*	0.621		[0.090, 2.021]	
	W-Actor	−0.465	−0.186	[−0.656, −0.273]	<0.001
	W-Partner	−0.106	−0.043	[−0.285, 0.077]	0.246
	W-*k*	0.229		[−0.149, 0.822]	

H, husband; W, wife; RT, responsibility thinking; PT, pro-setback thinking; FT, fate thinking; QMI, quality of marriage index.

**Figure 2:**
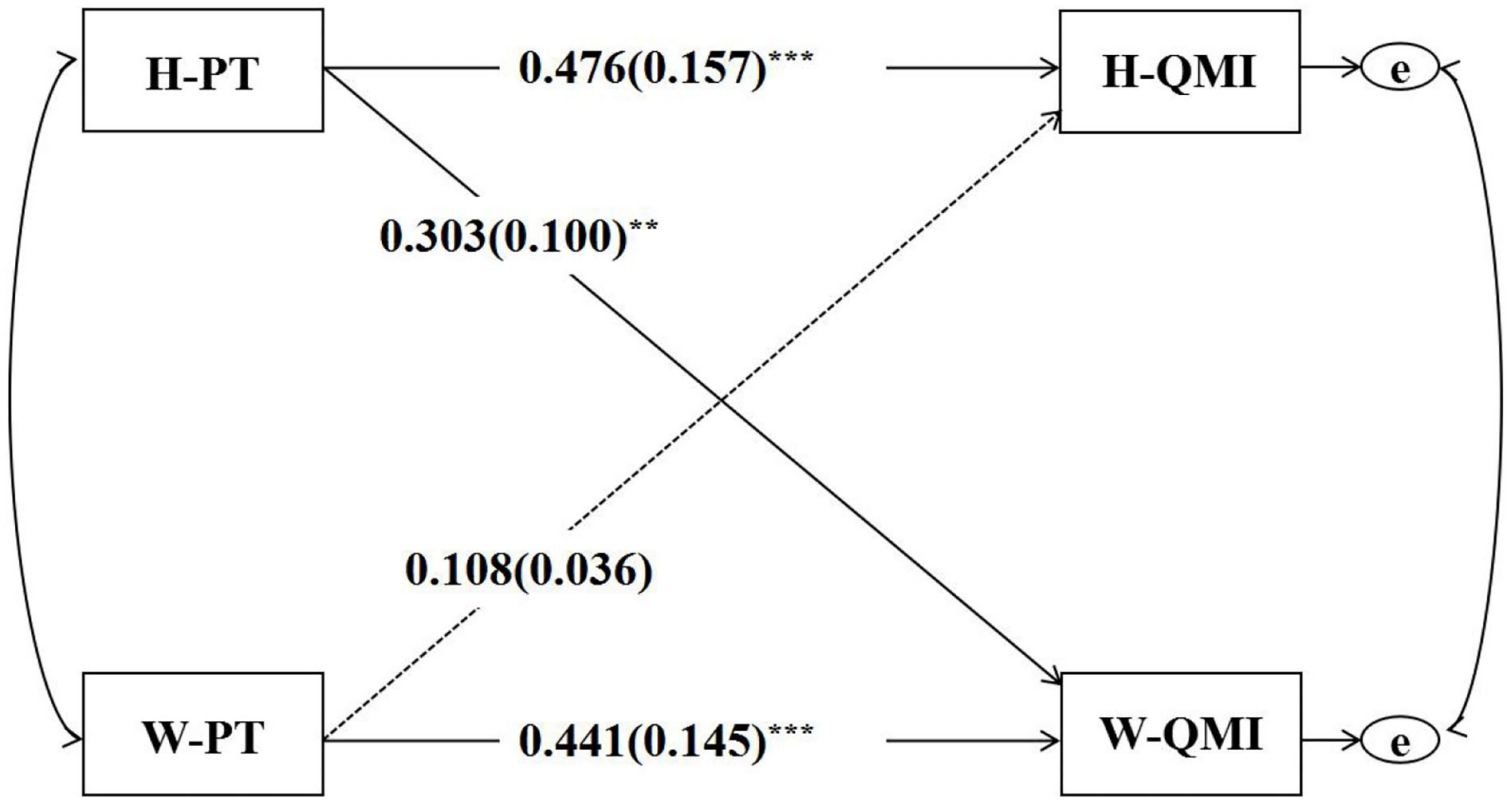
The APIM for PT's predictive effect on QMI. PT, pro-setback thinking; QMI, quality of marriage index; H, husband; W, wife; **p < 0.01, ***p < 0.001.

The APIM for the predictive effect of couples' pro-setback thinking on marital quality is shown in Figure 2, and the actor and partner effects for husbands and wives are presented in Table 2. When tested if the two actor effects were equal, the difference was found not to be statistically significant [*p* = 0.823, 95% CI (−0.27, 0.34)]. In addition, when tested if the two partner effects were equal, the difference was found not to be statistically significant [*p* = 0.186, 95% CI (−0.48, 0.09)]. Husbands' *k*-value was 0.227 with a 95% CI between −0.181 and 0.853 (0 was in the CI), suggesting the actor-only pattern. Wives' *k*-value was 0.686 with a 95% CI between 0.191 and 1.778 (1 was in the CI), suggesting the couple pattern. In addition, the two *k-*values showed no significant difference [*p* = 0.426, 95% CI (−1.71, 0.44)]. Whether the model was with distinguishable dyad members was further tested. Based on the results, χ^2^(15) was equal to 156.15 and *p* was lower than 0.001, suggesting that the predictive effect of husbands' and wives' pro-setback thinking on marital quality was statistically distinguishable dyad members by gender.

**Figure 3:**
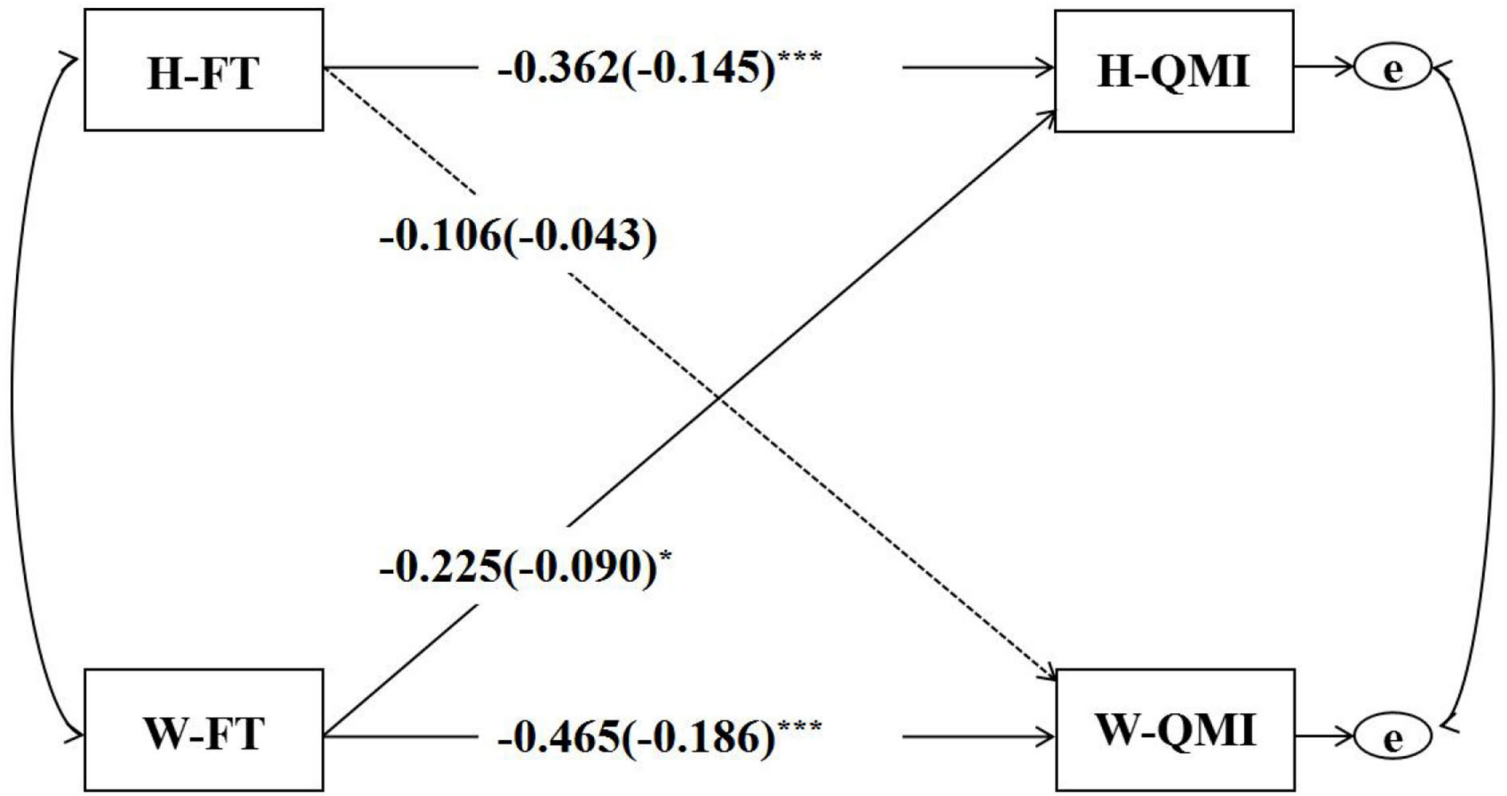
The APIM for FT's predictive effect on QMI. FT, fate thinking; QMI, quality of marriage index; H, husband; W, wife; *p < 0.05, ***p < 0.001.

The APIM for the predictive effect of couples' fate thinking on marital quality is shown in Figure 3, and the actor and partner effects for husbands and wives are presented in Table 2. When tested if the two actor effects were equal, the difference was found not to be statistically significant [*p* = 0.489, 95% CI (−0.19, 0.40)]. Besides, when tested if the two partner effects were equal, the difference was found not to be statistically significant [*p* = 0.407, 95% CI (−0.41, 0.16)]. Husbands' *k*-value was 0.621 with a 95% CI between 0.090 and 2.021 (1 was in the CI), suggesting the couple pattern. Wives' *k*-value was 0.229 with a 95% CI between −0.149 and 0.822 (0 was in the CI), suggesting the actor-only pattern. In addition, the two *k-*values showed no significant difference [*p* = 0.672, 95% CI (−0.54, 1.93)]. Whether the model was with distinguishable dyad members was further tested. According to the results, χ^2^(15) was equal to 174.92 and *p* was lower than 0.001, suggesting that the predictive effect of husbands' and wives' fate thinking on marital quality was statistically distinguishable dyad members by gender.

## Discussion

The studies pertaining to coping approaches and marital quality have always been the hotspot of marriage and family fields. Marital quality, as individuals' subjective feelings, is not static. Influenced by social culture, personality traits, interaction modes, attachment styles, and so forth, marital quality is always a dynamic process. Among a variety of factors, coping approaches play a vital role in affecting marital quality (56). People in the same cultural context may show certain common characteristics (15). As the mainstream culture of China, Confucianism has shaped the value judgment and behavioral patterns of Chinese people. Although an increasing number of scholars have realized that coping approaches have clear cultural characteristics (57), hitherto, no study has probed the relationship between Confucian coping approaches and marital quality. Particularly, no study has used dyadic data to investigate the relationship between the actor and partner effects of husbands' and wives' responsibility thinking, pro-setback thinking, and fate thinking on marital quality. Based on the APIM, the present study, with middle-aged and old couples as participants, investigated the predictive effect of Confucian coping thinking on individuals' own and their spouses' marital quality. At present, China has been facing the social problem of a rising divorce rate (58). Marital quality has been considered the most important predictor of marital stability (59). The results of the present study can be conducive to deepening the understanding of the relationship between Confucian coping thinking and marital quality, so as to provide theoretical reference for improving marital quality and reducing the divorce rate.

In the APIM for the predictive effect of responsibility thinking on marital quality, both husbands' and wives' responsibility thinking could significantly positively predict their own and their spouses' marital quality. Responsibility thinking is the premise of developing and maintaining interpersonal relationships. According to previous studies, responsibility can improve marital quality (60). To the authors' knowledge, the present study is the first one that investigated the relationship between responsibility thinking and marital quality and drew a similar conclusion. Responsibility's contribution to a marriage has been identified in both Chinese and western cultures, although in the two cultures, the emphasis and view on responsibility are not identical, and the connotation and manifestation of responsibility are also different. Confucianism regards the self as a kind of “relational self,” and believes that everyone should be responsible for others in a relationship, which requires individuals to actively improve their self-cultivation in order to better take their responsibility (61). As the most basic social relationship, marital relationships are considered a person's main responsibility (62). Both husbands and wives have the responsibility to jointly maintain the stability, sustainability, and harmony of marriage. Influenced by Confucianism's view on family, individuals attaching importance to marital harmony tend to voluntarily take family responsibility and even sacrifice their own interests (63).

In the APIM of the predictive effect of pro-setback thinking on marital quality, both husbands' and wives' pro-setback thinking could significantly positively predict their own marital quality, which was consistent with the hypothesis. However, only husbands' pro-setback thinking could positively wives' marital quality, which was not in line with the hypothesis. Based on previous studies, the predictive effect of couples' coping approaches on marital satisfaction is not identical (56). For instance, Bodnmann et al. found that husbands' positive coping approaches were positively correlated with couples' marital quality, while wives' positive approaches were only correlated with their own marital quality and were not correlated with their husbands' marital quality (46). Pro-setback thinking, as a sort of positive coping thinking, also shares the same predictive effect. Confucianism assumes that both men and women should take responsibility in line with their gender, identity, and status, and act accordingly. On the contrary, the cultivation of pro-setback thinking is more aimed at men. Confucianism regards “improving oneself,” “striving for progress,” and “daring to take responsibility” as important spiritual characteristics of a “gentleman” personality (64). Confucianism insists that men should hold a positive attitude toward the setbacks and hardships encountered in their careers and growth. In addition, Confucianism's concept of hierarchy is also reflected in marital relationships, assuming that women should be subordinate and obedient to men (65). Women's happiness is largely dependent on men's career achievements. Setbacks are considered the key to self-transcendence and success (31). Therefore, husbands' optimistic attitude in handling and overcoming setbacks means that they are more likely to succeed in the future, which can enhance the wife's evaluation of marital quality.

In the APIM of the predictive effect of fate thinking on marital quality, both husbands and wives had actor and partner effects, namely, both husbands' and wives' fate thinking could significantly negatively predict their own marital quality, which was consistent with the hypothesis. According to previous studies, fate thinking, different from responsibility thinking and pro-setback thinking, can negatively predict individuals' psychological health, emotional experience, and psychological resilience (27, 31). The present study also demonstrated in the marital relationship field that fate thinking was negative. Fate thinking shows individuals' inclination of attributing it to external uncontrollable factors when encountering stress. Individuals with higher fate thinking are more inclined to attribute the good or bad results to “fate,” and passively accept and comply with the “predetermined” results (66). The idea of “obeying fate” can cause uncontrollability and powerlessness. Individuals with high fate thinking usually take tolerant and passive approaches in coping with the confrontation and dissatisfaction in marriage, which may cause a decline in marital quality.

In the analysis of the partner effect, the present study only found that wives' fate thinking could negatively predict their husbands' marital quality, while husbands' fate thinking could not significantly predict their wives' marital quality, which was not completely consistent with the hypothesis. Fate is a complicated concept, comprising three dimensions: “fearing fate,” “obeying fate” and “utilizing fate.” Among them, “fearing fate” is the most negative attitude toward fate. Individuals fearing fate tend to believe that everyday issues have already been ordained by fate, and then passively accept them. In Confucianism's view on marriage, women have no free choice of their husbands, so their perceived marital quality is completely dependent on their predetermined husbands, which manifests in “fearing fate” (67). In other words, women would blame their marital miseries on the fate of failing to marry a good husband (68). Women's complaints about their husbands and marriage could negatively affect their own and their husbands' marital quality. In addition, different from the view of women, Confucianism emphasizes that “Junzi” (gentlemen) should “obey fate.” With the prerequisite of fate being unchangeable, “obeying fate” assumes that personal morality could be strengthened to realize, understand, and obey fate (69). Confucianism believes that a “Junzi” should always strengthen his self-cultivation, although it is a very difficult and long process from “fearing fate” to “obeying fate.” Therefore, men are more inclined to ascribe the negative effect of fate to their lack of self-cultivation, which properly justifies husbands' FT only negatively predicting their own perceived marital quality but failing to significantly predict their wives' marital quality.

Adopting the APIM, the present study investigated the relationship between Confucian coping thinking and marital quality. The present study has certain theoretical significance, as it is an expansion and supplement to the Vulnerability-Stress-Adaptation (VSA) Model and the family systems theory. In addition, the present study also has certain practical significance, as it can serve as a reference for marriage therapy and family intervention. From the perspective of culture, different and targeted measures can be developed to improve marital quality. Moreover, marital quality can also be enhanced by cultivating couples' responsibility thinking and pro-setback thinking and reducing their fate thinking. It is noteworthy that although Confucian coping thinking is relatively stable and significantly correlated with marital quality, individuals' coping approaches are by no means invariable, and the specific strategies shown in a specific context of stress are affected by internal and external factors (70). More specifically, the coping approaches adopted by couples in handling and solving various problems or stress in marriage are also related to both the thinking and context at that time (71). Hence, in the intervention of marital quality, the psychological features, interaction modes, family of origin, economic status, and other factors of couples also need to be investigated comprehensively, in addition to the role of Confucian coping thinking.

The present study also has some limitations. First, a cross-sectional survey was used in the present study, so the causality between Confucian coping thinking and marital quality is hard to be explained. A longitudinal survey needs to be conducted in future work to probe how Confucian coping thinking affects marital quality as time rolls on. Second, the data were collected by self-report measures, which may cause recall bias. Besides, China has the concept of “don't wash your dirty linen in public,” which possibly leads to the inaccuracy of the results under the Social Desirability Effect. Third, in the process of China's modernization, Chinese culture is characterized by diversity. Especially, younger people are deeply influenced by western culture (72). Consequently, in terms of Confucian coping thinking, as well as the attitude and evaluation of marital quality, young couples may show relatively huge differences from their middle-aged and old counterparts. Therefore, whether the results in the present study are applicable to young couples still needs more empirical evidence. Fourth, the sample was insufficient in representativeness, since participants were all recruited from college students' acquaintances based on convenient sampling and snowball sampling. Therefore, random sampling can be adopted in future work to further examine the relationship between Confucian coping thinking and marital quality.

## Data availability statement

The original contributions presented in the study are included in the article/supplementary material, further inquiries can be directed to the corresponding author.

## Ethics statement

The studies involving human participants were reviewed and approved by the Ethics Committee of Jilin International Studies University. The patients/participants provided their written informed consent to participate in this study.

## Author contributions

All authors listed have made a substantial, direct, and intellectual contribution to the work and approved it for publication.

## Funding

This study was supported by the Higher Education Research Project of Jilin Province (jgjx2021D149) and the Social Science Funding Support Project for PhDs and Youth of Jilin Province (2022C8).

## Conflict of interest

The authors declare that the research was conducted in the absence of any commercial or financial relationships that could be construed as a potential conflict of interest.

## Publisher's note

All claims expressed in this article are solely those of the authors and do not necessarily represent those of their affiliated organizations, or those of the publisher, the editors and the reviewers. Any product that may be evaluated in this article, or claim that may be made by its manufacturer, is not guaranteed or endorsed by the publisher.
